# Recollections of a Helmstetter Disciple

**DOI:** 10.3390/life13051114

**Published:** 2023-04-30

**Authors:** Alan C. Leonard

**Affiliations:** Department of Biological Sciences, Florida Institute of Technology, 150 W. University Blvd., Melbourne, FL 32952, USA; aleonard@fit.edu

**Keywords:** bacterial cell cycle, *oriC*, minichromosomes, DNA supercoiling, chromosome segregation

## Abstract

Nearly fifty years ago, it became possible to construct *E. coli* minichromosomes using recombinant DNA technology. These very small replicons, comprising the unique replication origin of the chromosome *oriC* coupled to a drug resistance marker, provided new opportunities to study the regulation of bacterial chromosome replication, were key to obtaining the nucleotide sequence information encoded into *oriC* and were essential for the development of a ground-breaking in vitro replication system. However, true authenticity of the minichromosome model system required that they replicate during the cell cycle with chromosome-like timing specificity. I was fortunate enough to have the opportunity to construct *E. coli* minichromosomes in the laboratory of Charles Helmstetter and, for the first time, measure minichromosome cell cycle regulation. In this review, I discuss the evolution of this project along with some additional studies from that time related to the DNA topology and segregation properties of minichromosomes. Despite the significant passage of time, it is clear that large gaps in our understanding of *oriC* regulation still remain. I discuss some specific topics that continue to be worthy of further study.

## 1. Introduction

My meeting with Charles Helmstetter was not planned. As much as I would like to say that it was my life-long dream to work in his lab, nothing could be further from the truth. Our meeting was completely accidental. I arrived at the Roswell Park Cancer Institute in Buffalo, New York wanting to be trained as a cancer research scientist. To me, that meant working with animals, or at the very least, tumor tissue. The idea of spending time in a lab that studied *E. coli* as a model system was unimaginable at the time. With a budding interest in nucleic acids, I sought out a faculty mentor whose laboratory was focused on the study of RNA polymerase activity in leukemic mice. My initial training in the lab was in enzyme purification, specifically the three forms of RNA polymerase from normal and leukemia virus-infected mouse spleens: mostly an experience of learning column chromatography in the cold room.

My limited expertise in the purification and handling of enzymes would turn out to be an important aspect of my introduction to Charles Helmstetter’s work and his laboratory group. Another was a fortuitous department reorganization at the institute. The lab I was working in was assigned to a newly organized Experimental Biology department, and while moving into new space was exciting, it also meant dealing with a new boss. The new department head was a well-respected biophysicist known for the exceptional quality of his research, but I knew little about him or his work. A bigger disappointment, at least in my mind, was that he worked on *E. coli*. Why would anyone at a cancer research institute study bacterial cells? With little interest in his research, I only hoped he was a benevolent leader, and I did my best to stay out of his way. It turned out that I was not successful at remaining anonymous for very long. Within the first year, our new head, Charles Helmstetter, came looking for some assistance and my faculty advisor volunteered me to lend a hand. This was not exactly a dream come true for me given my bad attitude, but at the time it seemed politically wise to provide the assistance, and perhaps I could quickly train one of Helmstetter’s grad students to replace me.

**Figure 1:**
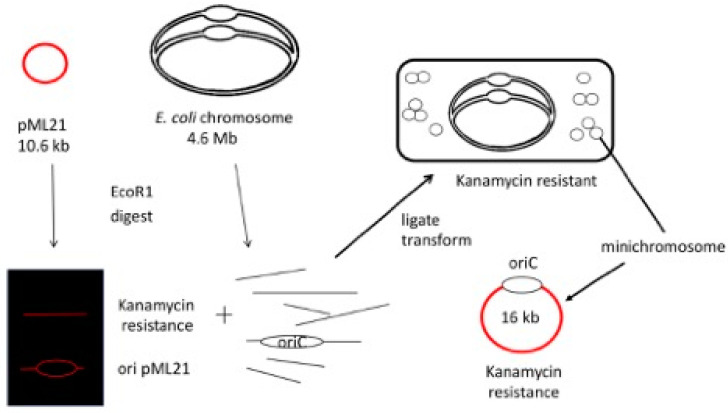
Scheme for construction of minichromosomes. Red color denotes plasmid DNA. Chromosomes are shown in a fast growth configuration used to increase the relative copies of *oriC*. See text for a description of the figure.

As initially described to me at a lab meeting with his group, the technical aspects of the project were straightforward: clone the replication origin (*oriC*) from the *E. coli* chromosome and construct an autonomously replicating miniature derivative: a minichromosome. Since studies of *oriC* on the chromosome were limited, particularly because it was an essential region, minichromosomes would be a useful tool. At the time, the location of *oriC* was known with respect to its nearby restriction endonuclease cut sites [1,2], and in theory, the pool of restriction fragments derived from the entire genome could be joined randomly at low concentrations with a non-replicating drug resistance marker isolated from a commonly available plasmid [3]. Only when the marker fragment was joined to *oriC* would it be capable of autonomous replication (see Figure 1).

Using current technology, this whole project could be completed in about a week, so it may not be obvious why the Helmstetter lab would need any assistance from me. The reader must remember, however, that this work was conducted over forty years ago, prior to the discovery of polymerase chain reaction, and required the brand-new technology of recombinant DNA, using enzymes that at the time were not yet commercially available. The restriction endonucleases and ligases had to be purified from over-producing *E. coli* strains before the project could even be started, and the DNA isolation protocols were not yet established in the lab. I was not intimidated by these roadblocks and remained hopeful that the project could be completed relatively quickly.

I retained my “why would a cancer researcher study *E. coli*” lack of enthusiasm and did not really appreciate why the cloned replication origin was such a prize. Perhaps sensing this, Charles insisted that I meet with him in his office so that he could explain why the project was important. I do not remember all the details of that meeting, nor can I say that I completely understood what he told me. What I do remember was that this was a transformative moment in my career. Charles described his seminal experimental work performed (with Steve Cooper) in the 1960s and explained how these experiments led to the development of the elegant model that describes the bacterial cell cycle: the I + C + D model. Although beyond the scope of this review, this work is beautifully presented in an accessible way in [4], and I urge anyone newly interested in this topic to start with this manuscript. Charles told me the story of the clever technology known as the baby machine, and how “backward” pulse labeling experiments revealed the cell cycle times of G1, S, and G2 (B, C, and D in *E. coli*). He also explained that the bacterial cell cycle was not a cycle at all, but a series of overlapping events; for example see [5,6]. Most impressive to me was that knowing the value of C + D, Charles could tell me exactly when new rounds of chromosome replication would initiate during the cell cycle in cultures growing at any rate. I had never heard anything like this before and the fact that he had worked out the laws for a growing cell (even an *E. coli* cell) was thrilling. I left Charles’ office truly enlightened and newly enthusiastic to become part of his lab group. I finally realized why studying *E. coli* was not only appropriate for cancer research, but essential to understand normal cell growth regulation. You could say that I became a true “Helmstetter disciple” from that moment onward.

## 2. Making *E. coli* Minichromosomes and Cell Cycle Analysis

**Figure 2:**
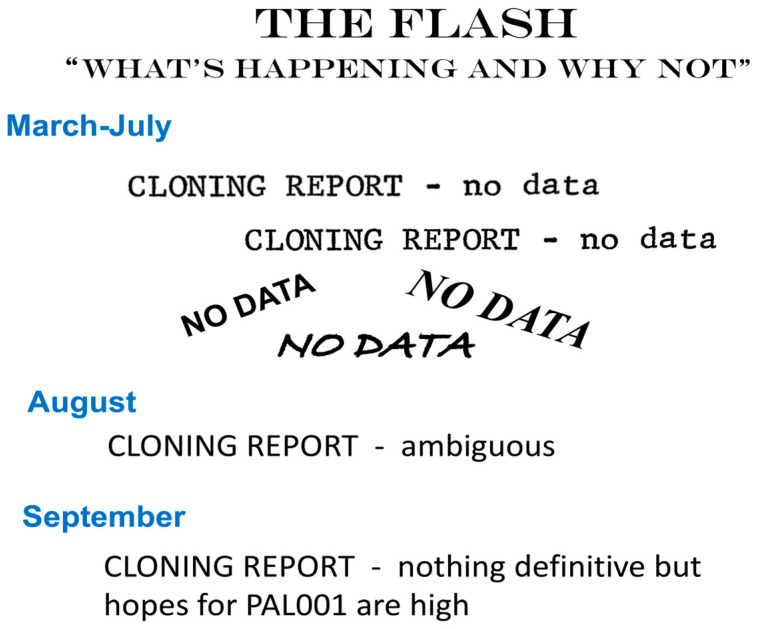
*OriC* cloning reports from the weekly lab newsletter “The Flash”. See text.

It turned out that cloning *oriC* was not as simple a task as I had hoped. A big stumbling block was my attempt to purify the non-replicating drug resistant fragment away from the plasmid’s own replication origin. This was achieved using gel electrophoresis to separate the two fragments and then purifying out the desired one. It was inefficient and subject to cross contamination. There were no kits available to speed the process, so I failed many times at this step and at the final step of cell transformation with the ligated chromosomal fragments. Unhappily, my repeated failures were noticed by one of Charles’ post-docs who, in an effort to be amusing, added a “cloning report” to his weekly tongue-in-cheek newsletter (The Flash) on lab group happenings. While I looked forward to his funny and often clever take on the events of the week, the weekly cloning report of “no progress” was not particularly uplifting (Figure 2).

When several interesting colonies finally appeared on a transformation plate and were shown to carry a new plasmid of the correct size expected for a minichromosome, I was horrified to observe that these plasmids were highly unstable in my *E. coli* host. This turned out to be due to the carry-over of some genes from the ATP synthetase operon adjacent to *oriC* on the chromosome, but until I was able to construct deletion derivatives, I grew cultures containing my first minichromosome (pAL1) at very high concentrations of antibiotic to kill off the plasmid-less segregants. Using 0.5 mg/mL kanamycin in the media seemed ridiculous at the time, but it allowed me to isolate enough minichromosome DNA for future experiments.

It is important to note that I was not the first to make an *E. coli* minichromosome. That honor went to others in the labs of Yuki Hirota and Walter Messer/Kaspar von Meyenberg, whose ground-breaking work provided the nucleotide sequence of *oriC* and began the difficult process of identifying the protein recognition motifs [7,8]. The enormous efforts of Bob Fuller and Jon Kaguni in Arthur Kornberg’s lab then produced an in vitro replication system that was pivotal to dissecting the chromosomal replication machinery [9].

Since our lab was focused on the regulation of DNA replication in living cells, our intent was to measure the replication of minichromosomes during the cell cycle. The obvious question was whether the minimal *oriC* region was sufficient for proper initiation timing during the cell cycle. The answer would not only reveal important features of the regulatory mechanism, but validate the minichromosome model for future studies, particularly those performed using in vitro systems. While we hoped that periodic minichromosome replication was retained, their moderately high copy number (10–20 copies per cell), despite their instability) did not seem compatible with the properly timed, once-per-cycle regulation of chromosomal *oriC*. We anticipated random replication but did not discount the possibility of periodic replication with initiation timed differently than the chromosome.

Rather than working with synchronously growing cells, we based our experimental design on the “backwards” baby machine approach Helmstetter and Cooper had used previously to study chromosome replication [5], since this would minimize artifacts caused by manipulating the cells. Since minichromosomes replicate in a matter of seconds, we believed that a minimally manipulated system would be important in distinguishing cell cycle-specific replication from random replication throughout the cell cycle. In this “backwards” procedure, exponentially growing cultures were pulse-radiolabeled with tritiated thymidine to label any replicating DNA. Then, the entire culture was transferred to a nitrocellulose membrane filter, the unincorporated label was washed out, and baby cells were eluted and collected at 1/10th generation intervals from the dividing cell population on the surface. The eluted cells contained the radiolabel incorporated into minichromosome and chromosomal DNA during the brief pulse label. The next trick was to develop a whole cell lysis protocol that was quantitative and free from the variable recovery artifacts caused when using the multi-step plasmid isolation methods available at the time. By examining many samples eluted over multiple generations of growth, we felt there was a good chance of obtaining a truthful result.

**Figure 3:**
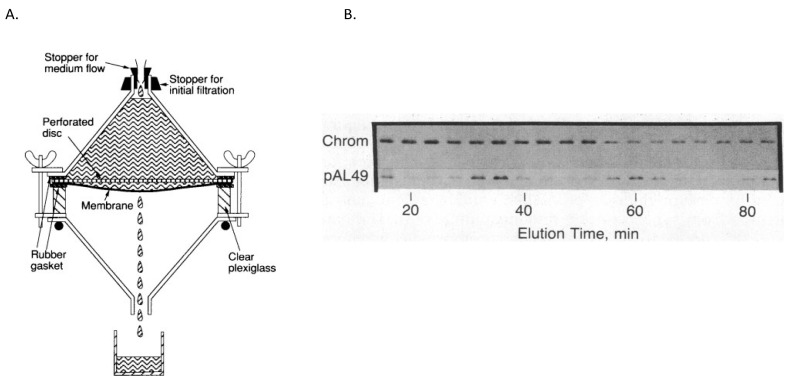
Baby machine analysis of minichromosome cell cycle replication. (**A**). Diagram of baby machine apparatus and sample collection. Cells growing exponentially were labeled with [^3^H]thymidine for 4 min, bound to a membrane filter, and eluted with glucose/Casamino acids minimal medium. (**B**). Electrophoretic separation of labeled chromosome and pAL49 minichromosome DNA from new daughter cells. Whole-cell lysates of new daughter cells in the effluent were subjected to agarose gel electrophoresis and fluorography. The radioactive bands corresponding to chromosomal and pAL49 DNA are shown for consecutive 4 min samples of the effluent. Exposure times to the x-ray films were 3 h for the chromosomal bands and 10 days for the minichromosome bands. Modified from reference [10].

**Figure 4:**
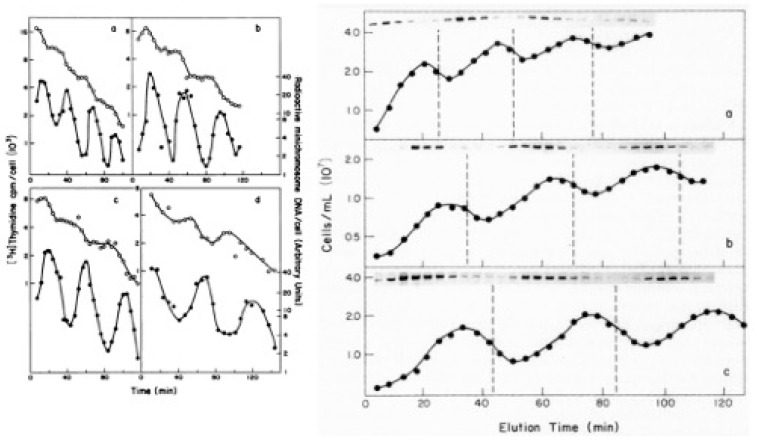
(Left panel) Timing of chromosome and pAL49 minichromosome replication during the division cycle. Exponential-phase cultures of *E. coli* B/r F26(pAL49) growing in glucose plus Casamino Acids (**a**), glucose plus six amino acids (**b**), glucose (**c**), or glycerol (**d**) were pulse-labeled and treated as described in the legend to Figure 3. The radioactivity per cell in minichromosome DNA (closed circles) and total radioactivity per cell (open circles) in newborn cells collected from the effluents of membrane-bound cultures are plotted at the midpoints of the 4 min collection intervals. Abrupt increases in radiolabel (reading right to left) indicate the time of initiation of chromosomal DNA replication. (Right panel). Minichromosome replication during the division cycle of *E. coli* B/r F(pAL49) growing at different rates. Cells growing exponentially in minimal medium containing glucose plus Casamino acids (**a**), glucose plus six amino acids (**b**), or glucose alone (**c**) were pulse-labeled with [^3^H]thymidine for 4 min, bound to a membrane filter, and eluted with minimal medium of the same composition. Whole-cell lysates of the newborn cells were treated as in Figure 3. Radioactivity corresponding to closed circular pAL49 minichromosome DNA is shown for consecutive 4 min samples of the effluent at each growth rate. The cell concentrations are also shown, and the vertical interrupted lines indicate the end of each generation of growth on the membrane. Modified from reference [11].

For every cell cycle sample collected over multiple generations of growth, chromosomes and minichromosomes were separated on agarose gels, which were processed, dried, and placed against X-ray film for an extended period of time. I then took the exposed films to a free-standing X-ray developing machine in one of the nearby clinics. The machine would emit many strange noises before the developed film would plop out into a plastic receptacle. I still recall our apprehension as Charles and I would stand there waiting to see each film emerge from the machine. These were often less than beautiful, with missing samples or streaky lanes, but these failures were completely forgotten when the films began to clearly show that minichromosomes were not only cell cycle specific replicons (see Figure 3 and [10]), but also initiated coincidently with the chromosome’s *oriC* in successive generations of growth and over a wide variety of growth rates; see Figure 4 [11]. I cannot adequately describe our excitement and how much these findings reshaped our future experiments, as well as our thinking about models for cell cycle regulation of *oriC*. It was also gratifying to see how enthusiastically our findings were accepted by our colleagues in the field. Of course, these studies required many trial and error experiments that extended well beyond my time as a graduate student, and most were conducted after I became a legitimate post-doctoral trainee in the Helmstetter lab. I had not only fallen in love with the science, but also with a doctoral student in the Pharmacology department (Julia Grimwade), who eventually became my wife and ultimately co-investigator in our own lab.

## 3. Searching for Cell Cycle-Specificity in Plasmid Systems

**Figure 5:**
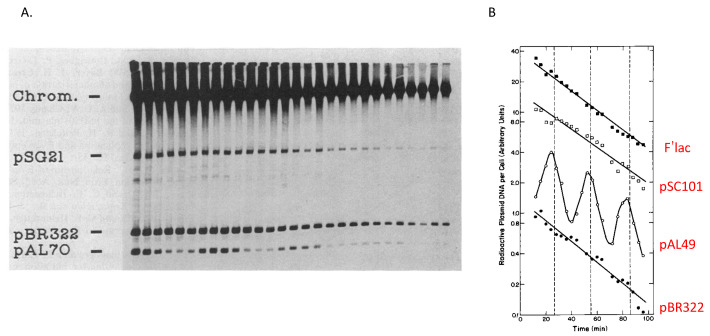
Cell cycle replication of various plasmids. (**A**). Fluorograph of radioactive plasmid DNA in newborn cells from the effluent of a membrane filter-bound culture of *E. coli* B/rF26 containing pSG21 mini-F, pBR322, and pAL70 simultaneously. Cells were grown, pulse-labeled, and prepared as in Figure 3. In this experiment, all lanes contained lysate from the same number of newborn cells. (**B**). Radioactive plasmid DNA in newborn cells from a membrane filter-bound culture of *E. coli* B/r F26 containing F’ lac, pSClO1, pAL49, and pBR322 simultaneously. Cells growing exponentially were pulse labeled and treated as described in Figure 3. Modified from [16].

With the clear cell cycle-specific replication pattern observed for minichromosomes, Charles and I turned our attention to other extrachromosomal replicons in *E. coli*. At the time, plasmid systems were commonly used as surrogates to study chromosome replication, and we were intrigued by the possibility that some plasmids would behave similarly to minichromosomes, particularly the low copy types such as F factors and the many plasmids whose replication origins interact with the chromosomal initiator protein DnaA; some examples are P1 [12], pSC101 [13], R1 [14], and mini F [15]. It seemed obvious that we should use minichromosomes as an internal control for cycle-specific replication, and our studies at the time uniquely included multiple compatible replicons co-inhabiting the same *E. coli* cell. The baby machine procedure to measure minichromosome replication during the cell cycle could be used unaltered, as long as we were careful to use plasmids of sizes that could be resolved from one another on agarose gels. While our efforts were limited to only the most prominent model systems (F, ColE1, pBR322, pSC101, and R1 derivatives), we were unable to identify any plasmid types that showed cell cycle-specific periodicity similar to minichromosomes (for example, see Figure 5, and [16]. Our F plasmid replication data from baby machine experiments was later analyzed using stochastics to reveal that the replication rate function increased monotonically over the cell cycle, with a rapid increase near cell division [17]. The resulting model is consistent with a replication control mechanism that is designed to force most plasmids to replicate before cells undergo division. Extending this model to the case of cell cycle-dependent replication requires additional, as yet unspecified control elements. Later experiments were extended to NR1 and P1 replicons [18]. OriP1 was able to initiate replication at all stages of the cell cycle with a slight periodicity observed in slower growing cells and NR1 plasmid replication was random during the cell cycle.

Some controversy about plasmid replication remains unresolved, since later studies from Steve Cooper’s lab were consistent with cell cycle specific replication for R6K, P1, F, and mini-F [19,20,21,22]. It is not clear why their results contrasted so dramatically with ours. Since all experiments were performed using baby machine selection, any differences in replication patterns must be due to differences in the *E. coli* strains, the post-processing of samples, and/or the assay of radiolabeled DNA.

We were intrigued to find that plasmids whose replication origins were known to interact with DnaA did not use this protein to couple their replication to the cell cycle. For these plasmids, DnaA availability might serve as a monitor of host metabolic activity, acting as either an on or off switch for plasmid replication depending on the plasmid type; for example [23,24]. This function would depend on the free availability of DnaA and not necessarily the ATP-bound state of the initiator. Our inability to identify cell cycle-specific plasmid replicons also raised the question of whether cell-cycle-specific or chromosome-coupled replication is ever beneficial for plasmids. Insights may come from bacteria containing two heterologous chromosomes (see Discussion).

## 4. A Sidestep into the Role of DNA Supercoiling in Minichromosome Regulation

During my time studying minichromosome replication, rapid advancements were being made in the study of bacterial regulation of DNA supercoiling; for example see reviews [25,26]. These studies intrigued me, and I began discussing DNA supercoiling regulation with Karl Drlica, who at the time was at the University of Rochester, just a short drive away from our lab in Buffalo. There were a number of mutant strains available with defective DNA gyrase (*gyrB* mutants) and topoisomerase 1 (*topA*), and examining the behavior of minichromosomes in increased and decreased supercoiling strains seemed like an interesting way to assess the supercoiling requirements for *oriC* function.

**Figure 6:**
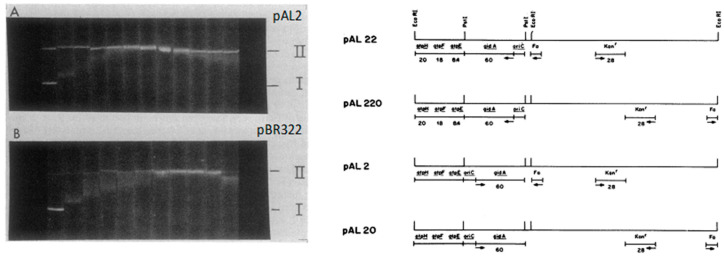
(Left panel) Dye titrations of pAL2 and pBR322 closed circular DNA. pAL2 (**A**) and pBR322 (**B**) DNA was isolated from JTT1 recA grown at 37 °C and electrophoresed in gels containing increasing concentrations of ethidium bromide. pAL2 and pBR322 were electrophoresed through 0.6 and 0.8% agarose, respectively. The concentrations of EtBr (in hundredths of micrograms per milliliter) from left to right are 0, 1, 2, 3, 4, 5, 6, 7, 8, 9, 10 and 11. (I) and (II) are supercoiled and relaxed-nicked circular DNA, respectively. (Right panel) Positions of promoter sequences and the direction of transcription on minichromosomes pAL2, pAL20, pAL22, and pAL220 (indicated by arrows). Only pAL20 and pAL22 were able to replicate in *E. coli* strains (*topA, gyrB*) with decreased supercoiling. Modified from [27].

We observed that minichromosomes were sensitive to DNA supercoiling activity and were very unstable in decreased supercoiling strains, in contrast to a variety of other plasmid types whose replication was unperturbed in these strains [27]. We also observed that minichromosomes had significantly lower superhelical density compared to other commonly studied plasmids (Figure 6). However, the stability of minichromosome replication was modulated by the arrangement of active transcriptional promoters on the plasmid (Figure 6). 

The roles of supercoiling and transcription-driven supercoiling activation of *oriC* were further characterized by others [28,29,30], and the relationship between transcription, supercoiling, and genome structure remains an active area of study; for example see [31,32,33]. However, it has yet to be determined whether the molecular requirements for *oriC* function differ under high or low supercoiling conditions in vivo.

## 5. Studying the Non-Random Segregation of Minichromosomes

**Figure 7:**
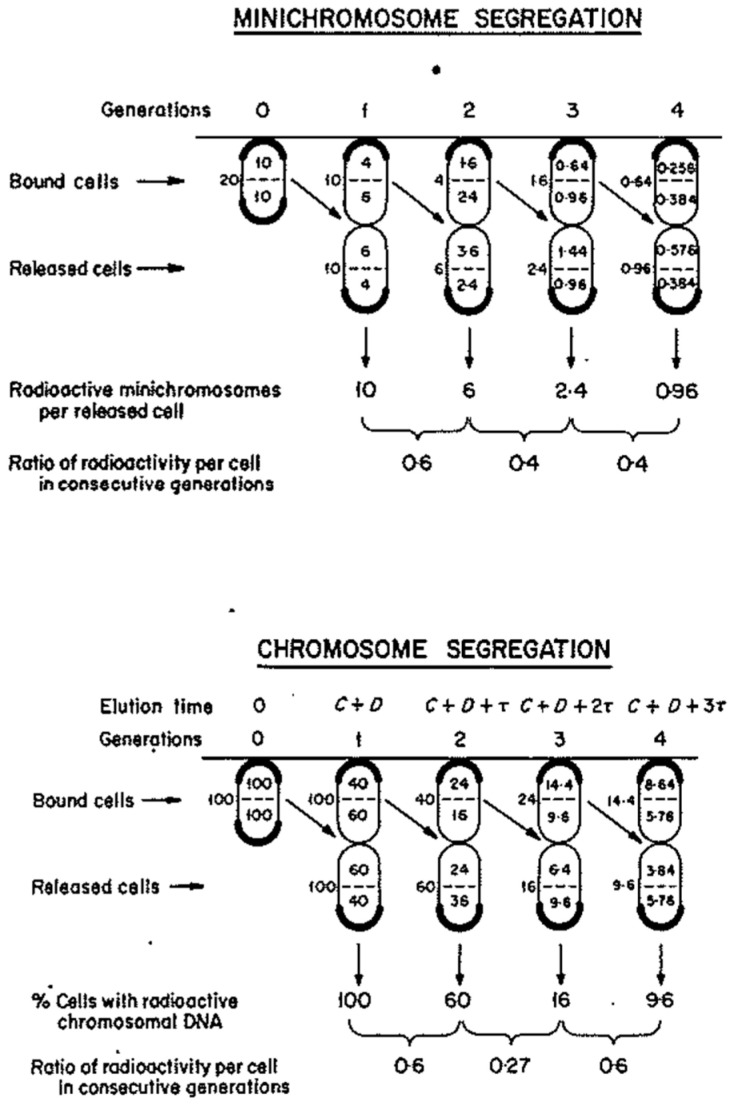
Segregation of minichromosomes and chromosomes during baby machine analysis. Theoretical segregation patterns of radiolabeled DNA are shown for bound (on the horizontal line) and released cells growing with a generation time of C + D minutes. Four successive generations are shown. Old poles lacking attachment sites are shown by thick lines. (Upper panel) Minichromosome segregation is shown with cells that initially contain 20 copies. Average copy numbers of labeled molecules are shown to the left of each cell and the internal distribution of copies is shown above and below the dotted line representing the division septum. The radiolabeled copies in released cells are also shown. (Lower panel) Chromosome segregation. Radiolabeled chromosomal strands (assuming 2 chromosomes in the initially bound cell) are shown in a similar fashion to panel A. Modified from [37].

*E. coli* chromosome segregation is nonrandom, despite the presence of an equipartition mechanism that ensures both daughter cells inherit complete genomes. In studies using the “backward” baby machine method, measurement of pulse-radiolabeled chromosomal DNA among progeny cells revealed that label does not segregate with the expected 50–50 distribution, but rather displays a distinctive, growth-media dependent, non-random distribution in successive generations; for more detail see [34,35,36]. It was difficult to envision a model that explained this mode of segregation, but our best ideas at the time were based on a mechanism determined by cell geometry, with chromosomes behaving as though they were restricted to particular cell locations. The simplest model to impart these restrictions was to envision an intracellular distribution of attachment sites for *oriC* that did not include the existing cell poles (e.g., the poles are dead for chromosome attachment); see Figure 7 and [37] for more detail. Lateral cell envelope growth would provide new attachment sites, but their distribution would remain asymmetric. The non-randomness of segregation would also be dependent on the size of the poles as well as the growth rate, consistent with experimental observations [35,36,38].

Was minichromosomes segregation (despite the lack of an equipartition mechanism) compatible with a model based on *oriC* attachment? Although the ratios of radiolabel released in consecutive generations were not identical to the chromosome, minichromosomes did indeed segregate non-randomly with a distinct pattern that was compatible with the model; see Figure 7 [37,39]. However, evidence for these hypothetical attachment sites for *oriC* remains sparse [40,41]. The activation of initiator protein DnaA by membrane acidic phospholipids is better understood, reviewed in [42,43,44], but it remains to be determined whether DnaA plays any role in chromosome segregation (see Discussion).

## 6. Discussion

While considerable effort was made over the intervening decades to understand the regulatory mechanisms for bacterial chromosome replication and segregation, the projects I describe above remain incomplete, because fundamental questions remain unanswered. This is undoubtably due to the complexity of the mechanisms involved, but also to limitations in technology. However, the recent dramatic shift from batch culture studies to single cell analysis has provided the bacterial cell cycle field with a new path forward and many interesting new models for bacterial size regulation, as well as the relationship between chromosome replication and cell division; for example see [45,46,47,48,49,50,51,52]. It is worth noting that these models are not necessarily in agreement with one another, adding a new level of intrigue to the field.

For studies of the initiation step of chromosome replication, analysis of the molecular machinery in single cells is particularly challenging. New approaches will be needed, but whatever technology is applied, the mechanism must have the following properties: (1) it must be able to accommodate once-only initiation from each copy of *oriC* during each cell cycle, (2) it must have the ability to synchronously initiate replication from many copies of *oriC* as a system that does not count origins, (3) it must trigger DnaA-dependent initiation events at the correct time during the cell cycle over a wide range of growth conditions, and (4) it must accommodate the ordered assembly of DnaA multimeric complexes on each copy of *oriC*, reviewed in [53]. Few models are able to satisfy these requirements, but, in my opinion, the initiation titrator model [54], which is now over 30 years old, still remains the front-runner. Yet, even this gold standard may require some tweaking to accommodate fast as well as slow growth conditions; see [55,56]. Finally, the amazing diversity of *oriC* nucleotide sequences obtained from a wide range of microbial types suggests that, despite conservation of the DnaA initiator protein, there are many ways to assemble a functional initiation complex (orisome). Bacteria may use fundamentally different schemes to regulate chromosome replication as best suits the environment of each organism.

Is plasmid replication coupled to the cell division cycle in any bacterial type? Uncoupled replication control provides the best opportunity for plasmid survival and the plasmid-encoded negative regulatory element(s) required for autonomous replication [57] are not compatible with cell cycle-specific plasmid replication. However, the domestication of plasmids has occurred in bacteria with two heterologous chromosomes [58,59], and studies should reveal how plasmid-derived, secondary chromosome replication is controlled during the cell cycle. While it is too early to know whether multiple mechanisms for plasmid domestication exist, studies of *Vibrio cholerae’s* chromosome 2 reveal a highly complex regulation for both cell cycle specificity and copy control that remains to be completely understood [60,61,62,63,64].

The relationship between DNA supercoiling, transcription, and the regulation of chromosomal replication origins in bacteria continues to be an under-explored area of research. However, it was recently demonstrated that during the stringent response (such as during nutrient limitation), global reduction of transcription by ppGpp alters DNA supercoiling sufficiently to prevent replication initiations from *oriC* [32]. This finding suggests that other mechanisms may also regulate the assembly of replication origin nucleoprotein complexes by local or global alterations of chromosome supercoiling; see related discussion in [65]. Dissecting these networks will be a complex task, particularly if the transcriptional activity is also coupled to the architecture of the chromosomes and the density of genes during replication; see reviews [33,66].

The topic of non-random segregation of *E. coli* chromosomes remained essentially dormant for over 20 years, but some recent publications indicate that it has been re-discovered [67,68,69]. Of particular interest is the finding that MukBEF and MatP proteins are involved in nonrandom segregation [67]. MukBEF is the *E. coli* equivalent of the structural maintenance of chromosomes (SMC) complexes found in all cell types, which organize chromosomes and are required for their faithful segregation, reviewed in [70]. MukBEF complexes have a distinctive folded shape that allows movement of DNA stands for regulation of nucleoid shape and chromosome decatenation [71]. MukBEF interact with multiple binding sites around the chromosome, but MatP is able to displace MukBEF from its DNA binding sites within the terminus region [72,73].

There are several ways that MukBEF and MatP might play roles that are compatible with our aforementioned *oriC* attachment model for nonrandom chromosome segregation. MukBEF sites are prominently clustered around *oriC* [74], and the replication origins interact with MukBEF complexes in a self-organizing system [75,76]. Any viable model must include the dynamic assembly and disassembly of these complexes as the cell cycle proceeds. MatP’s ability to displace MukBEF and direct it towards (or away) from *oriC* may provide an opportunity for specifically timed assemblies.

What about attachment of *oriC* to internal surface sites? MukBEF complexes may be part of the direct attachment mechanism, but I prefer a model whereby MukBEF produces a particular structure in the *oriC* region that is necessary for attachment. It is reported that MukBEF is capable of DNA loop extrusion [77], and a MukBEF-produced loop might allow *oriC* to be accessible for surface attachment. A novel MukBEF-dependent mechanism for nonrandom chromosome replication may also exist that does not require any attachment of *oriC* to cell surface sites. Hopefully, this will become a future model to test.

## Data Availability

Data sharing is not applicable.
